# Prevalence, predictors, and prognosis of tricuspid regurgitation following permanent pacemaker implantation

**DOI:** 10.1371/journal.pone.0235230

**Published:** 2020-06-26

**Authors:** Jiwon Seo, Dae-Young Kim, Iksung Cho, Geu-Ru Hong, Jong-Won Ha, Chi Young Shim

**Affiliations:** Division of Cardiology, Severance Cardiovascular Hospital, Yonsei University College of Medicine, Yonsei University Health System, Seoul, Korea; University Medical Center Groningen, University of Groningen, NETHERLANDS

## Abstract

This study aimed to investigate the prevalence and clinical significance of lead-related tricuspid regurgitation (TR) in patients with permanent pacemaker (PM). A total of 2,533 patients who underwent permanent PM implantation between January 2008 and December 2017 in a single center were retrospectively reviewed. Among them, 429 patients who underwent transthoracic echocardiography within 90 days before implantation and were followed up at least 3 months after PM implantation were included. Patients who had pre-existing grade 3 or 4 TR, had a single atrial lead, or had undergone tricuspid valve surgery before PM implantation were excluded. Occurrence of PM-related TR (PMTR) was defined as worsening of TR by at least 2 grades on follow-up echocardiography. Cardiovascular outcomes were defined as the composite of cardiovascular death and hospitalization for heart failure. During the median follow-up of 855 days, 42 (9.8%) patients had PMTR and 86 (20.0%) presented with cardiovascular outcomes. In the multivariate logistic regression analysis, the presence of atrial fibrillation (hazard ratio [HR]: 2.07, 95% confidence interval [CI]: 1.27–4.09, p = 0.037]) and history of open-heart surgery (HR: 3.34, 95% CI: 1.68–6.68, p<0.001) were independently associated with PMTR. Patients with PMTR showed significantly higher cardiovascular events than those without (45.2 vs. 17.3%, log-rank p<0.001). Furthermore, PMTR was independently associated with the primary outcome (HR: 2.45, 95% CI: 1.43–4.22, p = 0.001). In conclusion, the occurrence of TR in patients with permanent PM is not uncommon. PMTR is associated with atrial fibrillation, the history of open-heart surgery, and poorer cardiovascular outcomes.

## Introduction

Implantation of permanent pacemakers (PM) has gradually increased because of either prolonged life expectancy or increased incidence of cardiac surgery [1, 2]. Tricuspid regurgitation (TR) is recognized as one of the lead-related complications [1, 3, 4]. PM-related TR (PMTR) can occur due to direct leaflet damage, such as leaflet perforation, lead entanglement, or lead adherence from fibrosis [2, 5]. In addition, TR can be aggravated through annular dilatation and chronic right ventricular (RV) dysfunction regardless of the mechanical interference to the valve by the electrode [6].

Several previous studies have demonstrated that moderate or severe TR occurred at a significant higher rate in patients with PM and was associated with higher mortality and heart failure-related hospitalization than that in risk-matched cohorts [7–9]. Although clinical implications of PMTR are largely concordant among previous studies[7–9], diverse predictors of PMTR have been proposed in each study. Alternative strategies to pace the heart without passing through the tricuspid valve, such as the placement of a coronary sinus pacing lead, surgical epicardial placement of leads, and leadless pacing systems, are currently available [10, 11]. Furthermore, echocardiography guidance at the time of PM implantation might be helpful to avoid acute leaflet damage by a transvenous lead in high-risk patients for PMTR. Therefore, we sought to investigate the prevalence, predictors, and prognosis of PMTR in a large, single-center registry involving a wide spectrum of patients who underwent permanent PM implantation in this study.

## Materials and methods

### Study population

**Fig 1:**
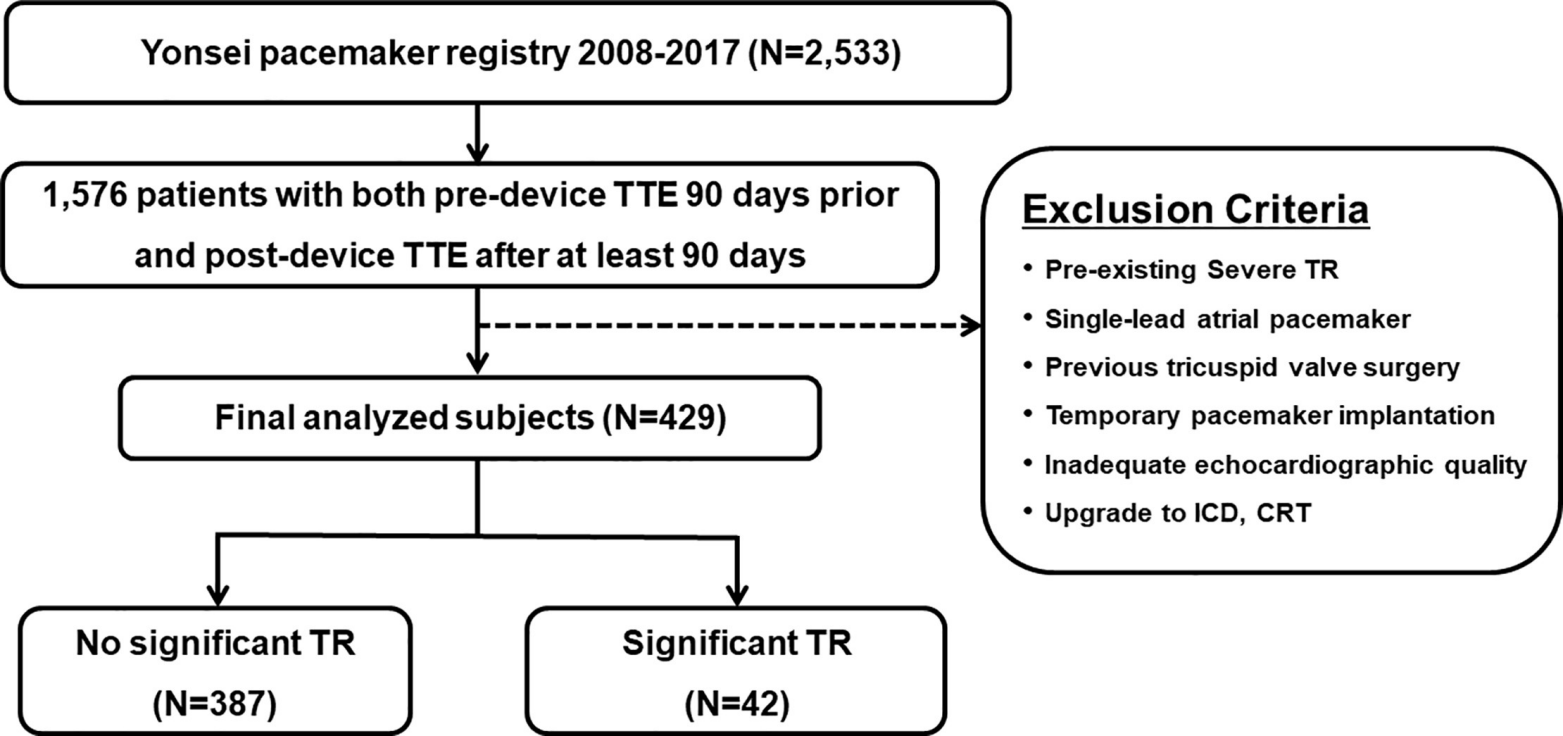
Flowchart of the study.

A total of 2,533 patients who underwent de novo permanent PM implantation at a single tertiary center between January 2008 and December 2017 were included in this analysis. Among them, 429 patients who underwent transthoracic echocardiography within 90 days before implantation and underwent follow-up at least 3 months after PM implantation were included. Data on baseline patient characteristics, implantation procedure, device characteristics and setting, and all follow-up visits were retrospectively reviewed. Indications for device implantation, based on international guidelines, were sick sinus syndrome and advanced atrioventricular block in PM recipients. We excluded patients who had grade 3 or 4 TR before PM implantation; were treated with a single-lead atrial PM, implantable cardioverter defibrillator, or cardiac resynchronization therapy; had a history of tricuspid valve repair; had an inadequate quality of echocardiographic data for assessment of RV function and degree of TR; or had undergone a temporary PM implantation (Fig 1). PMTR was defined as TR worsened by at least 2 grades on post-implantation echocardiography during the follow-up period. Patients were divided into two groups according to the occurrence of PMTR: Group 1 (n = 387, no PMTR) and Group 2 (n = 42, PMTR). The study was approved by the Institutional Review Board of Yonsei University Health System (approval number: 4-2020-0032), and it complied with the Declaration of Helsinki. As registry-based retrospective study and the data were analyzed anonymously, it did not require informed consent from the study subjects.

### Echocardiography

Standard two-dimensional and Doppler measurements were performed following the American Society of Echocardiography guidelines [12]. TR severity was graded semi-quantitatively using colored and continuous wave Doppler data using a multi-parametric approach [13, 14]. RV systolic dysfunction was evaluated by two-dimensional echocardiography. RV area measurements were obtained from the apical four-chamber focused RV view at end-diastole and end-systole, and RV fractional area change was calculated as the ratio between the difference at the end-diastolic and end-systolic RV areas and the end-diastolic area [12]. The TV annulus dimension (TVAD) was measured as the end-diastole in the apical four-chamber view and then indexed by the body surface area (BSA) [13]. The RV systolic pressure was calculated from the maximum velocity of the TR jet according to the modified Bernoulli equation [12]. Echocardiographic data were carefully reviewed by two experienced cardiologists who were blinded to the clinical data.

### Follow-up and outcomes

Patients were regularly scheduled to visit the PM clinic every 6 months after PM implantation. Follow-up data were obtained by reviewing medical records. If patients did not visit on the scheduled day, outcome data were assessed via telephone interview. The primary outcome was defined as a composite of cardiovascular death and hospitalization due to heart failure during the follow-up period.

### Statistical analysis

Continuous variables were expressed as mean ± standard deviation. Categorical variables were expressed as number (percentage). Between-group comparisons were performed using standard χ^2^ tests for categorical variables and paired *t*-tests for continuous variables. Univariate and multivariate logistic regression analyses were performed. Comparison of worsening of TR, mitral regurgitation (MR) and RV systolic pressure (RVSP) in tertile of ventricular pacing percentage were performed using the standard χ^2^ tests and analysis of variance (ANOVA) test. Pearson's correlation coefficient was used to test the linear relation between the percentage of ventricular pacing and RVSP. Survival curves were constructed using the Kaplan–Meier method, and comparisons were made using the log-rank test. All statistical analyses were performed using SPSS Statistics software version 25.0 (IBM, Armonk, NY, USA); *p*-values < 0.05 were considered statistically significant.

## Results

### Baseline characteristics

Among 429 patients, 42 (9.8%) had PMTR. Clinical and echocardiographic characteristics of the study population, patients without PMTR, and those with PMTR are summarized in Table 1. Patients with PMTR tended to be older and of the female sex (without statistical significance). Patients with PMTR had a significantly higher incidence of previous open-heart surgery than those without PMTR. Regarding PM data, patients with PMTR showed a higher incidence of VVI mode although the V pacing percentage and indications for PM implantation were comparable. In terms of echocardiographic characteristics, patients with PMTR showed smaller RV end-diastole area and RV end-systole area than those without PMTR, although other echocardiographic variables were comparable, including left heart size and function, TVAD, RV systolic pressure, and initial TR grades. The smaller RV area in patients with PMTR seems to be associated with fewer cases of grade 1 or 2 TR in that group than in the group without PMTR, owing to the criteria for dividing the two groups.

**Table 1:** Baseline characteristics.

	Total (n = 429)	No PMTR (n = 387)	PMTR (n = 42)	*P*-value
**Demographic data**				
Age, years	67.0 ± 12.8	66.7 ± 13.0	70.3 ± 10.4	0.078
Male sex, n (%)	176 (41.0)	164 (42.4)	12 (28.6)	0.118
Body mass index, kg/m^2^	24.2 ± 3.5	24.2 ± 3.5	23.9 ± 3.5	0.580
Hypertension, n (%)	256 (59.7)	226 (58.4)	30 (71.4)	0.142
Diabetes mellitus, n (%)	104 (24.2)	91 (23.5)	13 (31.0)	0.380
Atrial fibrillation, n (%)	131 (30.5)	114 (29.5)	17 (40.5)	0.195
CAD, n (%)	53 (12.4)	47 (12.1)	6 (14.3)	0.878
Open-heart surgery, n (%)	71 (16.6)	57 (14.7)	14 (33.3)	0.004
MV surgery	21 (4.9)	16 (4.1)	5 (11.9)	0.066
AV surgery	43 (10.0)	35 (9.0)	8 (19.0)	0.075
CABG	8 (1.9)	6 (1.6)	2 (4.8)	0.389
Others	11 (2.6)	10 (2.6)	1 (2.4)	>.999
**Pacemaker data**				
V pacing percentage, %	61.1 ± 42.5	61.5 ± 42.8	57.5 ± 40.3	0.571
Indication				
AV node disease, n (%)	264 (61.5)	238 (61.5)	26 (61.9)	>.999
Sinus node disease, n (%)	165 (38.5)	149 (38.5)	16 (38.1)	>.999
Mode				
DDD, n (%)	348 (81.1)	322 (83.2)	26 (61.9)	0.002
VDD, n (%)	7 (1.6)	7 (1.8)	0 (0.0)	0.812
VVI, n (%)	25 (5.8)	17 (4.4)	8 (19.0)	< .001
**Echocardiographic data**				
LVEF, %	65.6 ± 9.6	65.7 ± 9.5	65.2 ± 10.2	0.770
LA volume index, ml/m^2^	43.3 ± 17.8	43.3 ± 17.6	43.7 ± 19.9	0.902
RVEDA, cm	18.8 ± 7.3	19.0 ± 7.6	17.0 ± 4.2	0.008
RVESA, cm	10.2 ± 3.7	10.3 ± 3.8	9.3 ± 2.8	0.048
RVFAC, %	45.8 ± 9.0	45.8 ± 9.1	45.0 ± 8.9	0.568
TVAD, mm	29.5 ± 4.6	29.5 ± 4.6	29.5 ± 4.0	0.951
TVAD/BSA, mm/m^2^	18.1 ± 3.1	18.0 ± 3.1	18.3 ± 2.9	0.531
RVSP, mmHg	33.2 ± 12.6	33.4 ± 13.0	31.5 ± 9.1	0.224
Tricuspid regurgitation				0.398
No, n (%)	287 (66.9)	255 (65.9)	32 (76.2)	
Grade 1, n (%)	110 (25.6)	102 (26.4)	8 (19.0)	
Grade 2, n (%)	32 (7.5)	30 (7.8)	2 (4.8)	

PMTR, pacemaker-related tricuspid regurgitation; CAD, coronary artery disease; MV, mitral valve; AV, aortic valve; CABG, coronary artery bypass graft; AVB, Atrioventricular block; LVEF, left ventricular ejection fraction; LA, left atrium; RVEDA, right ventricular end-diastole area; RVESA, right ventricular end-systole area; TVAD, tricuspid valve annular diameter; BSA, body surface area; RVSP, right ventricular systolic pressure.

### Predictors for PMTR

Table 2 shows the factors associated with the development of PMTR. In the multivariate logistic regression analysis, the presence of atrial fibrillation (hazard ratio [HR] 2.07, 95% confidence interval [CI] 1.27–4.09, p = 0.037) and history of open-heart surgery (HR 3.34. 95% CI 1.68–6.68, p < 0.001) were independently associated with PMTR. Among echocardiographic variables, TVAD/BSA tended to be associated with PMTR, but the statistical significance was marginal (HR 1.12, 95% CI 0.99–1.26, p = 0.054). The presence of grade 1 or 2 TR on pre-implantation echocardiography did not increase the risk of PMTR compared to the absence of TR. The results of subgroup analysis of multivariate Cox proportional hazard ratios for PMTR according to the history of open-heart surgery are shown in Table 3. Comparison of worsening of TR, MR and RVSP in tertile of ventricular pacing percentage were presented in Table 4. There were no significant relationships between increase of ventricular pacing percentage and worsening of TR or MR. On the contrary, RVSP was correlated with percentage of ventricular pacing in Pearson’s correlation coefficient (R = 0.154, p = 0.001).

**Table 2:** Multivariate Cox proportional hazard ratios for pacemaker-related tricuspid regurgitation.

	HR (95% CI)	*P*-value
Age	1.03 (0.99–1.06)	0.072
Male sex	0.67 (0.32–1.37)	0.271
Atrial fibrillation	2.07 (1.27–4.09)	0.037
Open-heart surgery	3.34 (1.68–6.68)	<0.001
V pacing percentage	1.00 (0.99–1.01)	0.901
LVEF	1.01 (0.97–1.04)	0.789
LA volume index	1.00 (0.98–1.02)	0.893
RVEDA	0.93 (0.86–1.02)	0.114
RVFAC	0.99 (0.95–1.02)	0.444
TVAD/BSA	1.12 (0.99–1.26)	0.054
RVSP	1.00 (0.97–1.03)	0.893
Tricuspid regurgitation		
No	Ref	Ref
Grade 1	0.47 (0.20–1.11)	0.086
Grade 2	0.43 (0.08–2.24)	0.314

HR, hazard ratio; CI, confidence interval; LVEF, left ventricular ejection fraction; LA, left atrium; RVEDA, right ventricular end-diastole area; RVESA, right ventricular end-systole area; TVAD, tricuspid valve annular diameter; BSA, body surface area; RVSP, right ventricular systolic pressure.

**Table 3:** Subgroup analysis of multivariate Cox proportional hazard ratios for PMTR according to the history of open-heart surgery.

	No open-heart surgery (n = 358)		Open-heart surgery (n = 71)	
	HR (95% CI)	P value	HR (95% CI)	P value
Age	1.03 (0.98–1.05)	0.395	1.07 (0.98–1.05)	0.045
Male sex	0.84 (0.34–2.07)	0.698	0.61 (0.34–2.07)	0.529
Atrial fibrillation	1.91 (0.79–4.62)	0.151	3.24 (0.87–12.06)	0.080
V pacing percentage	1.00 (0.99–1.01)	0.885	1.00 (0.98–1.02)	0.948
LVEF	0.99 (0.95–1.04)	0.827	1.03 (0.95–1.04)	0.349
LA volume index	0.99 (0.96–1.01)	0.313	0.99 (0.96–1.01)	0.617
RVEDA	0.92 (0.83–1.02)	0.101	1.02 (0.84–1.24)	0.832
RVFAC	0.99 (0.95–1.04)	0.717	1.01 (0.94–1.09)	0.840
TVAD/BSA	1.12 (0.96–1.30)	0.158	1.28 (0.98–1.65)	0.065
RVSP	1.01 (0.97–1.06)	0.554	1.02 (0.95–1.10)	0.557
TR				
No	Ref	Ref	Ref	
Grade 1	0.16 (0.03–0.77)	0.022	1.60 (0.33–7.71)	0.559
Grade 2	0.01 (0.08-inf)	0.997	1.54 (0.16–15.12)	0.712

HR, hazard ratio; CI, confidence interval; LVEF, left ventricular ejection fraction; LA, left atrium; RVEDA, right ventricular end-diastole area; RVESA, right ventricular end-systole area; TVAD, tricuspid valve annular diameter; BSA, body surface area’ RVSP, right ventricular systolic pressure.

**Table 4:** Comparison of worsening of TR, MR and RVSP in tertile of ventricular pacing percentage.

	V pacing <30% (n = 147)	V pacing 30 to 99.5% (n = 133)	V pacing >99.5% (n = 149)	*P*-value
PMTR, n (%)	14 (9.5)	17 (12.8)	11 (7.4)	0.311
Worsening of MR, n (%)	23 (15.6)	24 (18.0)	17 (11.4)	0.282
RV systolic pressure, mmHg ± SD	30.7 ± 9.8	34.8 ± 14.1	34.4 ± 13.4	0.011

V pacing, Ventricular pacing percentage; PMTR, pacemaker-related tricuspid regurgitation; MR, mitral regurgitation; RV, right ventricle.

### Impact of PMTR on clinical outcomes

**Fig 2:**
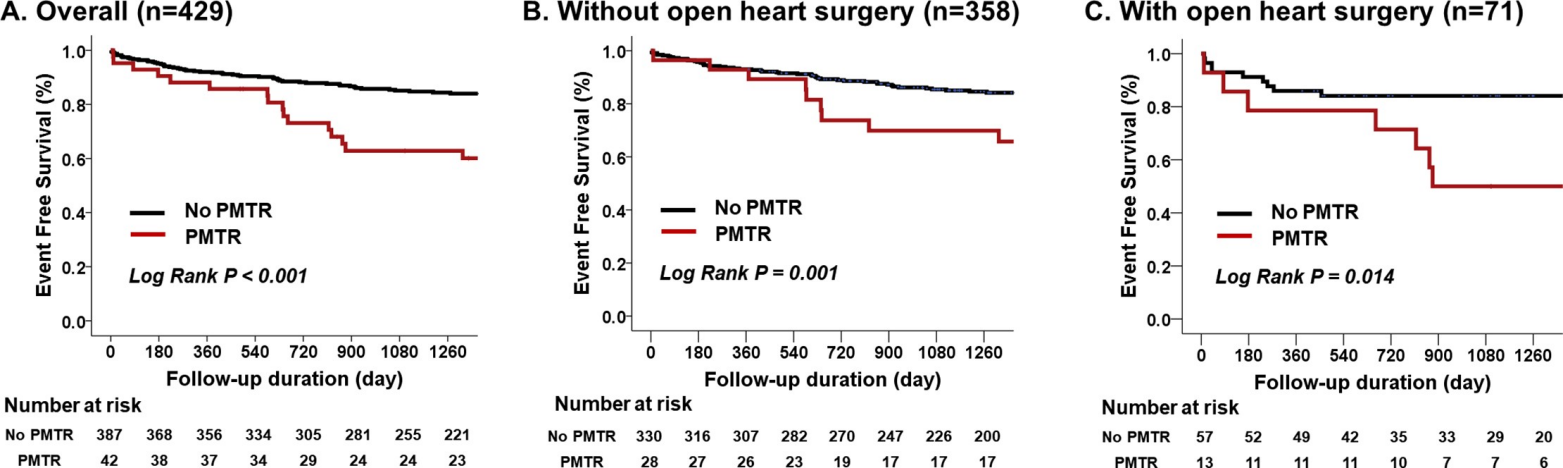
Kaplan–Meier curves for the primary outcome-free survival according to the presence of pacemaker-related tricuspid regurgitation A. overall patients, B. patients without previous open-heart surgery, and C. patients with previous open-heart surgery.

During the median follow-up of 855 days, patients with PMTR showed significantly higher cardiovascular events than those without (45.2% vs. 17.3%, log-rank p<0.001) (Fig 2A). PMTR was independently associated with primary outcomes (HR: 2.45, 95% CI: 1.43–4.22, p = 0.001) even after controlling for age; sex; comorbidities including coronary artery disease, atrial fibrillation, and history of open-heart surgery; and echocardiographic variables including LV ejection fraction and LA volume index (Table 5). In the subgroup analysis according to the history of open-heart surgery, the clinical impact of PMTR was consistent in patients without a history of open-heart surgery (Fig 2B, log-rank p = 0.002, HR 2.44, 95% CI 1.28–4.62, p = 0.007, Table 6) and in those with a history of open-heart surgery (Fig 2C, log-rank p = 0.014, HR 1.43, 95% CI 1.48–4.33, p = 0.012, Table 6).

**Table 5:** Multivariate Cox proportional hazard ratios for primary outcomes.

	HR (95% CI)	*P*-value
Age	1.05 (1.03–1.08)	<0.001
Male sex	1.19 (0.77–1.85)	0.437
CAD	1.14 (0.64–2.05)	0.656
Atrial fibrillation	1.23 (0.78–1.96)	0.377
Open-heart surgery	0.86 (0.48–1.53)	0.602
LVEF	0.96 (0.94–0.98)	<0.001
LA volume index	1.00 (0.99–1.01)	0.707
PMTR	2.45 (1.43–4.22)	0.001

CAD, coronary artery disease; LVEF, left ventricular ejection fraction; LA, left atrium; PMTR, pacemaker-related tricuspid regurgitation.

**Table 6:** Subgroup analysis of the multivariate Cox proportional hazard ratios for primary outcomes according to the history of open-heart surgery.

	No open-heart surgery (n = 358)		Open-heart surgery (n = 71)	
	HR (95% CI)	P value	HR (95% CI)	P value
Age	1.06 (1.03–1.09)	< .001	1.07 (1.03–1.11)	0.623
Male sex	1.39 (0.84–2.28)	0.199	2.85 (1.25–6.50)	0.614
CAD	1.10 (0.57–2.15)	0.770	0.47 (0.13–1.65)	0.717
Atrial fibrillation	1.52 (0.92–2.50)	0.101	1.28 (0.56–2.96)	0.258
LVEF	0.97 (0.94–0.99)	0.005	0.96 (0.93–0.99)	0.028
LA volume index	1.01 (0.99–1.02)	0.155	1.00 (0.97–1.02)	0.433
PMTR	2.44 (1.28–4.62)	0.007	1.43 (1.48–4.33)	0.012

CAD, coronary artery disease; LVEF, left ventricular ejection fraction; LA, left atrium; PMTR, pacemaker-related tricuspid regurgitation.

## Discussion

The primary findings of this study were as follows: 1) the prevalence of PMTR was 9.7% in this study population during a median follow-up of 855 days; therefore, it was not uncommon; 2) the occurrence of PMTR was associated with atrial fibrillation and a history of open-heart surgery; and 3) PMTR was associated with poorer cardiovascular outcome. Therefore, we would suggest that echocardiographic surveillance of PMTR should be performed in patients who underwent PM implantation, especially in those with atrial fibrillation or a history of open-heart surgery.

The results of this study are generally concordant with those of previous studies on the poor prognostic impact of PMTR [7–9]. However, the study population of each study is notably different. Höke et al. demonstrated that significant lead-induced TR is associated with poor long-term prognosis in 287 patients who underwent Implantable cardioverter defibrillator (ICD) or pacemaker implantation. They included 191 patients with ICD and excluded patients who underwent cardiac valve surgery. Therefore, the baseline LV ejection fraction of the study was 39 ± 14% because of a large proportion of patients with ICD implantation for primary or secondary prevention [9]. In contrast, the baseline LV ejection fraction in this study was 66 ± 10%, as we excluded patients who underwent ICD implantation or cardiac resynchronization therapy. Moreover, 16.6% of patients had a history of open-heart surgery, and of these, the majority were cardiac valve surgeries in the mitral or aortic position. Delling et al. also revealed that permanent PM-related significant TR was not a benign phenomenon but was associated with an increased mortality risk compared to that in patients with permanent PM without significant TR [9]. The study did not exclude patients with pre-existing grade 3 or 4 TR before PM implantation, and information regarding the presence of atrial fibrillation at baseline was lacking. Another study by Al-Bawardy et al. also did not exclude patients with pre-existing grade 3 or 4 TR from their study, and 62% of their study population had ICDs [7]. Therefore, this study provides further information regarding the prevalence, predictors, and prognosis of PMTR by including only patients with permanent PM in a wide spectrum of underlying disease including patients with history of open-heart surgery.

The etiology and mechanism of PMTR are not fully elucidated. Earlier studies mainly showed direct damage to TV leaflet or subvalvular apparatus during pacemaker lead implantation can lead to PMTR by perforation of TV leaflet, laceration, or injury of chordae tendineae [15–17]. However, more recent studies have demonstrated that mechanical interference of TV leaflet or subvalvular structures which resulted in incomplete coaptation of TV [3, 5]. Another mechanism of lead-related PMTR is fibrotic response and adhesion of lead and TV leaflet. One study reported that lead adherence to the TV leaflet occurred in 14 out of 41 patients with severe TR due to a permanent pacemaker or ICD [2]. Pacing-related TR also one of the possible mechanism of PMTR. Since LV dyssynchrony and left bundle branch block is an established cause of mitral regurgitation [18], RV dyssynchrony due to permanent pacing is considered can lead to a worsening of TR [6]. In this study, atrial fibrillation and a history of open-heart surgery were found to be independent predictors of PMTR. This finding suggested that the mechanism of PMTR in this population is likely to involve secondary TR, including interference of TV leaflet coaptation, lead adherence, or pacing-related TR rather than primary TR related with direct leaflet damage by the PM lead. Moreover, the larger TVAD/BSA on baseline echocardiogram before PM implantation tends to be associated with PMTR after adjusting for the RV area and presence of atrial fibrillation, which also supports the suggested mechanism.

With the increase in the aging population and recent advances in surgical and transcatheter intervention, many patients who underwent permanent PM implantation had atrial fibrillation and a history of valve procedure. Conduction abnormality requiring permanent PM implantation are frequent and associated with an increased risk of heart failure-related hospitalization and lack of functional improvement and reverse remodeling after either transcatheter or surgical aortic valve implantation [19–21]. Data regarding the prognostic impact of PM implantation after valve intervention are somewhat controversial. A few studies demonstrated no prognostic impact of PM implantation after valve implantation [19, 22]; however, some studies showed worse outcome in patients who received permanent PM implantation [21, 23]. These discrepancies may possibly be related to the involvement of the occurrence of PMTR between the PM implantation and clinical outcome. In this study, the clinical implication of PMTR was also significant in the subgroup of patients with a history of open-heart surgery.

This study has several limitations. First, this study was retrospectively designed, resulting in an inherent potential limitation. Patients who did not undergo follow-up echocardiography were excluded, and the interval after PM implantation in each patient was not preset in real clinical practice. Therefore, the prevalence of PMTR may possibly be underestimated. Second, a few studies have demonstrated that three-dimensional echocardiography might be better for the assessment of lead-TV interaction [24, 25], but we did not perform an analysis using three-dimensional echocardiography findings. Therefore, the mechanism of PMTR could not be determined in detail. Third, although one of the possible mechanism of pacing related TR is RV dyssynchrony, we did not include RV dyssynchrony analysis due to unavailable echocardiographic data for the analysis. However, we assumed that the main findings of the study are not substantially affected by RV dyssynchrony because percentage of ventricular pacing was not significantly associated with increasing of TR.

In conclusion, worsening of TR is not uncommon in patients with permanent PM and is associated with the presence of atrial fibrillation and a history of open-heart surgery. PMTR is independently associated with poor cardiovascular outcome. Thus, baseline and regular follow-up echocardiographic surveillance should be performed after PM implantation, especially in patients with atrial fibrillation and a history of open-heart surgery.
